# Integrative Metabolic-Flux Platform for Analysis, Contextualization, and Targeting (IMPACT): bridging untargeted and targeted flux analysis

**DOI:** 10.1093/bioinformatics/btaf591

**Published:** 2025-11-13

**Authors:** Collin Starke, Janneke Hendriks, Judith Wahrheit, Frank Fluitman, Jules Beekwilder, Florence Miramella Schempp, Karsten Hiller

**Affiliations:** Department of Bioinformatics and Biochemistry, Technische Universität Braunschweig, Niedersachsen, Braunschweig 38106, Germany; BASF Metabolome Solutions GmbH, Berlin 10589, Germany; Computational Biology, BASF SE, Ludwigshafen am Rhein 67056, Germany; Isobionics BV, Geleen 6167RD, The Netherlands; Isobionics BV, Geleen 6167RD, The Netherlands; Industrial Biotechnology, BASF SE, Ludwigshafen am Rhein 67056, Germany; Department of Bioinformatics and Biochemistry, Technische Universität Braunschweig, Niedersachsen, Braunschweig 38106, Germany

## Abstract

**Summary:**

The “Integrative Metabolic-Flux Platform for Analysis, Contextualization, and Targeting” (IMPACT) is a comprehensive, fully modular platform designed for both targeted and untargeted metabolomics analysis in stable-isotope labeling experiments. It facilitates the accurate calculation of mass isotopomer distributions (MIDs) and the annotation of unknown metabolites with a contextualization algorithm, addressing the challenges in metabolomics research. IMPACT integrates an entire preprocessing pipeline for LC-MS data, including peak picking, feature grouping, and peak filling, along with advanced features for isotope detection, MID calculation, and its core feature contextualization, enabling metabolite integration into biological pathway networks. The platform supports various file formats and offers user-friendly online access, making it accessible for researchers seeking to elucidate metabolic pathways and networks with precision and reliability.

**Availability and implementation:**

IMPACT is implemented in Python 3.9 and R 4.3.2, with a front-end in Javascript utilizing the Cytoscape.js library for data visualization. It is available as a docker container and can be accessed online at https://impact.bioinfo.nat.tu-bs.de, providing a user-friendly interface for metabolomics data analysis.

## 1 Introduction

Stable-isotope labeling has become an essential technique in metabolomics research for studying metabolic pathways and networks (Buescher *et al.* 2015). However, the accurate calculation of mass isotopomer distributions (MIDs) and the identification of unknown metabolites in stable isotope labeling experiments are crucial for obtaining reliable and meaningful results (Hiller *et al.* 2013, Nakabayashi and Saito 2020). Metabolomics and stable-isotope experiments are typically classified into two separate strategies: targeted and non-targeted/untargeted approaches. Both approaches have distinct advantages and disadvantages; targeted strategies shine with their precision and reliability but only cover a limited predefined set of metabolites. Untargeted strategies, on the other hand, offer the potential to detect novel essential features that could elucidate pathway and network knowledge (Ribbenstedt *et al.* 2018). Nevertheless, untargeted metabolomics faces significant challenges in accurately identifying metabolites due to the complexity of processing workflows and the limitations of identification databases and libraries (Schrimpe-Rutledge *et al.* 2016).

For LC-MS preprocessing, widely used tools include the R package xcms (Smith *et al.* 2006), the web platform XCMS Online (Tautenhahn *et al.* 2012), MZmine 3 (Schmid *et al.* 2023), and MS-DIAL (Tsugawa *et al.* 2015).

Beyond desktop pipelines, several web platforms partially support workflows for profiling data of stable-isotope labeling experiments. XCMS Online provides cloud LC-MS preprocessing, MetaboAnalyst offers web-based raw LC-MS processing and downstream analysis (Pang *et al.* 2024), and the Galaxy-based Workflow4Metabolomics provides a preprocessing ecosystem (Giacomoni *et al.* 2015). While X13CMS and DIMet can detect isotopic enrichment locally and in Galaxy respectively, they are unable to contextualize stable-isotope labeling patterns (Huang *et al.* 2014, Galvis *et al.* 2024). To our knowledge, no web solution converts raw LC-MS data into corrected MIDs and subsequently contextualizes those MIDs via similarity networks.

For this purpose, we developed the “Integrative Metabolic-Flux Platform for Analysis, Contextualization, and Targeting” (IMPACT), a fully modular LC-MS preprocessing and stable-isotope contextualization platform.

## 2 Features

**Figure 1.**
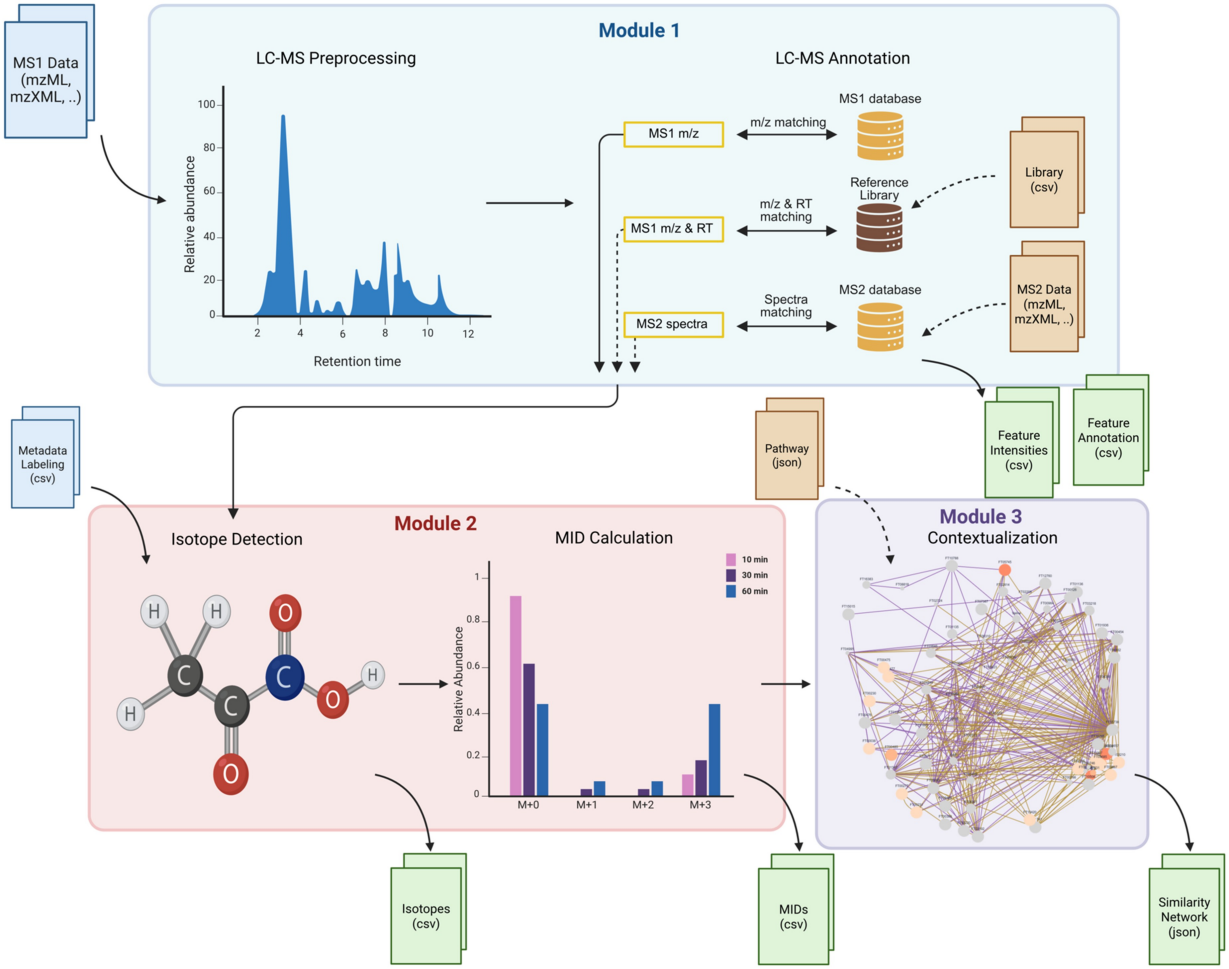
Overview of the IMPACT workflow. IMPACT comprises three modules: Module 1 (LC-MS preprocessing and annotation), Module 2 (isotope detection and MID calculation), and Module 3 (contextualization). File icons denote required inputs and outputs for each module and dashed arrows indicate optional paths. Module 1 currently supports only LC-MS (open formats mzML, mzXML, mzData); Modules 2 and 3 are instrument-agnostic and can start from feature intensity and annotation CSV tables for Module 2 or MID CSV tables for Module 3. The figure was created with BioRender.

Our tool, the “Integrative Metabolic-Flux Platform for Analysis, Contextualization, and Targeting,” addresses these challenges by offering a comprehensive set of features for targeted and untargeted metabolomics analysis. IMPACT fully supports LC-MS data across all three modules and supports already preprocessed GC-MS features for modules 2 and 3 through table inputs. IMPACT extends our NTFD (Hiller *et al.* 2013) and MIA (Weindl *et al.* 2016) software, which are capable of MID calculation from raw GC-MS data, and adds LC-MS preprocessing capabilities. Its modular design enables this versatility, which includes non-targeted isotope detection, Mass Isotopomer Distribution (MID) calculation, and contextualization of unknown metabolites, applicable across different mass spectrometry techniques (see Fig. 1).

IMPACT encompasses a complete preprocessing pipeline for LC-MS data utilizing the xcms R package (Smith *et al.* 2006), facilitating peak picking, retention time alignment, feature grouping, and peak filling. This pipeline automatically processes uploaded raw LC-MS files in the open formats mzML, mzXML, and mzData, allowing users to adjust parameters to fit their specific analysis needs. Following preprocessing, IMPACT provides three separate annotation modes: MS1 accurate mass matching, which is always enabled, and the two optional steps reference library mapping, and MS2 spectral library matching using the R package MetaboAnnotation (Rainer *et al.* 2022). Users can enable or disable library- and MS2-based matching independently. By default, MS1 annotation is performed, which matches each feature’s accurate mass to an adduct sum formula in an open-source MassBank database (Horai *et al.* 2010). For the second annotation option, we map previously measured standards on the same instrument to unknown features in the processed experiment, requiring measured standard retention time and spectral mass values. The final annotation option, MS2 annotation, involves uploading MS2 files. We process these files, align them with the original MS1 data, and match them against spectra in the MassBank MS2 database to annotate the MS1 features. Upon completing preprocessing and annotation, users can download feature intensity and annotation flat files as CSV files or proceed with further isotope detection. All preprocessing and annotation steps are independent of isotope labeling and therefore report both labeled and unlabeled features.

In the next module, isotope detection and MID calculation require at least one labeled condition. For isotope detection, IMPACT uses a modified algorithm from the X13CMS package (Huang *et al.* 2014). This step involves analyzing feature intensity and annotation data (generated in the previous step) alongside sample information to accurately detect isotopes based on mass differences and intensity patterns in labeled and unlabeled samples. The pipeline then calculates MIDs for the detected isotopes, which determines mass isotopomer distributions by correction with the unlabeled samples. Users can filter these results with various parameters, including minimum fraction of enrichment in labeled samples, maximum labeling in unlabeled samples, and others, which users can find in the documentation, along with explanations for all available options. Finally, both isotope detection and MID calculation results are available in CSV format and can be downloaded or used for the final step, contextualization.

In general, MIDs of metabolites in the same metabolic pathway are closely related (Weindl *et al.* 2016, Dudek *et al.* 2020). Calculating the similarity between known and unknown compounds can put unknown metabolites into context. For that purpose, IMPACT utilizes a contextualization algorithm to calculate similarity networks of all processed data. Users only need the previously generated MID data or a single CSV file with MIDs from any other source (and other descriptive information) to calculate a similarity network across multiple experiments and conditions. We also enable the mapping of metabolite MIDs onto reference pathways in Cytoscape JSON format. Users can analyze the contextualized network in IMPACT or download it directly. There are two analysis modes, “Unknown” and “Targeted.” Unknown mode creates a force-directed graph of all nodes, which can be filtered based on similarity scores, number of connections, and more. Users can inspect quantification, MID data, and statistical variabilities between experiments for quantity and labeling amount. “Targeted” mode maps specified metabolites onto the uploaded pathway based on an identifier the user can provide and customize. This minimalistic view makes it easy to spot differences in crucial pathway metabolites. Like in “Unknown” mode, users can highlight the nearest unknown neighbors of each pathway metabolite and compare quantification and MID data.

## 3 Implementation

IMPACTs back-end for preprocessing and data analysis is implemented in Python 3.9 and R 4.3.2. The front end is implemented in Javascript with the Cytoscape.js (Franz *et al.* 2016) library for visualization. IMPACT and it’s documentation is available as a docker container and online at https://impact.bioinfo.nat.tu-bs.de.

## 4 Conclusion

In conclusion, the “Integrative Metabolic-Flux Platform for Analysis, Contextualization, and Targeting” (IMPACT) bridges the gap between untargeted and targeted data processing of stable-isotope labeling experiments. With its modular approach, LC-MS data can be fully processed using the application pipeline, or other preprocessed data (regardless of experiment, measurement, or processing setup) can be used for isotope detection, MID calculation, and contextualization of unknowns within biological pathway networks. IMPACT is a user-friendly, accessible online tool that makes it easy for anyone to analyze untargeted metabolomics data.

## Data Availability

The IMPACT source code is accessible at https://github.com/CollinStark/impact. The source code is archived at https://doi.org/10.5281/zenodo.17044266.
